# Draft Genome Sequence of *Microbacterium* sp. Strain LEMMJ01, Isolated from Antarctic Ornithogenic Soil

**DOI:** 10.1128/genomeA.00672-17

**Published:** 2017-07-20

**Authors:** Júnia Schultz, Yuri Alves Pinheiro de Souza, Maria Cristina Pinheiro Pereira Reis Mansur, Alane Beatriz Vermelho, Fábio Faria da Mota, Alexandre Soares Rosado

**Affiliations:** a Laboratory of Molecular Microbial Ecology, Microbiology Institute, Federal University of Rio de Janeiro, Rio de Janeiro, Brazil; b BioInovar Laboratory, Microbiology Institute, Federal University of Rio de Janeiro, Rio de Janeiro, Brazil; c Oswaldo Cruz Institute, Fiocruz, Rio de Janeiro, Brazil

## Abstract

We report here the 3,637,012-bp draft genome sequence of *Microbacterium* sp. strain LEMMJ01, isolated from ornithogenic soil from King George Island, Antarctica. The total number of genes presented in the draft genome sequence was 3,553, and the total number of coding sequences was 3,497. In addition, genes related to the production of terpene and carotenoids were revealed.

## GENOME ANNOUNCEMENT

The genus *Microbacterium* is a member of the phylum *Actinobacteria*, a Gram-positive, aerobic, and heterotrophic bacterium (1, 2). It is found in different habitats, having been described in sponges, deep-sea sediments, humans, and polar environments (3–6). *Microbacterium* species have biotechnological importance, such as in biosurfactant production, bioremediation (7), and the application of their natural pigments, such as carotenoids, in the food, cosmetics, textiles, and pharmaceutical industries (8, 9).

We sequenced the draft genome sequence of *Microbacterium* sp. strain LEMMJ01, isolated from Antarctic ornithogenic soil collected close to the Arctowsky Polish Station near an Adelie penguin nest (62°09ʹ790ʺS; 58º27ʹ687ʺW) in Admiralty Bay, King George Island, Antarctica. The soil processing protocol was described in a previous work (10), and single colonies of the psychrophilic bacteria were isolated on LB medium after growing at 12°C for 48 h. The isolated colonies were then grown in liquid LB medium (12°C for 48 h), and their genomic DNA was extracted using the FastDNA SPIN kit for soil (MoBio) and quantified using a Qubit fluorometer (Thermo Fisher Scientific). The Nextera XT DNA library kit was used to build paired-end 250-bp libraries, which were sequenced on an Illumina MiSeq. Assembly was performed by MR DNA (Shallowater, TX) using NGen version 12 from DNAStar, and the coding DNA sequences (CDSs) were predicted and annotated using two tools, the NCBI Prokaryotic Genome Automatic Annotation Pipeline (PGAAP) and the Rapid Annotations using Subsystems Technology (RAST) server (11).

The draft assembly of *Microbacterium* sp. LEMMJ01 resulted in 3,637,012 bp, with 300× coverage and 70.20% GC content, present in 44 contigs, with an *N*_50_ of 335,663 bp. The total number of genes presented in the draft genome sequences was 3,553, and the total number of coding sequences was 3,497. In addition, 46 tRNAs and 56 rRNAs were predicted in the complete genome.

The results revealed, by PGAAP, that carotenoid biosynthesis genes were localized as a cluster that included genes encoding C50 carotenoid epsilon cyclases. Genes related to the production of terpenes were detected with antiSMASH (12), highlighting the biotechnological interest in this strain. The presence of resistance genes was verified by ResFinder 2.1 (13), with no positive results. Further analyses will be carried out with the genome sequences of the psychrophilic bacterium in order to better understand the biotechnological applications originating from the production of carotenoids at low temperatures by *Microbacterium* sp. LEMMJ01.

### Accession number(s).

This whole-genome sequence project has been deposited at DDBJ/ENA/GenBank under the accession no. NCTV00000000. The version described in this paper is version NCTV01000000.
